# The Charcot foot: a pictorial review

**DOI:** 10.1186/s13244-019-0768-9

**Published:** 2019-08-05

**Authors:** Andrea B. Rosskopf, Christos Loupatatzis, Christian W. A. Pfirrmann, Thomas Böni, Martin C. Berli

**Affiliations:** 1 Radiology, Balgrist University Hospital, Forchstrasse 340, 8008 Zurich, Switzerland; 2 Faculty of Medicine, University of Zurich, Zurich, Switzerland; 3 Radiology, Spital Maennedorf, Asylstrasse 10, 8708 Maennedorf, Switzerland; 4 Orthopedic Surgery, Balgrist University Hospital, Forchstrasse 340, 8008 Zurich, Switzerland

**Keywords:** Charcot foot, Imaging, Osteomyelitis, MRI, Radiographs

## Abstract

Charcot foot refers to an inflammatory pedal disease based on polyneuropathy; the detailed pathomechanism of the disease is still unclear. Since the most common cause of polyneuropathy in industrialized countries is diabetes mellitus, the prevalence in this risk group is very high, up to 35%. Patients with Charcot foot typically present in their fifties or sixties and most of them have had diabetes mellitus for at least 10 years. If left untreated, the disease leads to massive foot deformation. This review discusses the typical course of Charcot foot disease including radiographic and MR imaging findings for diagnosis, treatment, and detection of complications.

## Key points

- X-rays may be normal during early stage of Charcot foot
- MRI should be done with large field of view covering the entire foot
- MRI can be used for early diagnosis, monitoring of disease activity and complications
- Acute MRI findings include bone marrow edema, soft tissue edema, and subchondral fractures
- Chronic MRI findings include subchondral cysts, joint destructions, joint effusion, and bony proliferations

## Annotation regarding wording of planes of foot imaging in this review

Hindfoot: sagittal = parallel to long axis of metatarsal bones; coronal = perpendicular to long axis of metatarsal bones; axial = perpendicular to long axis of the tibia.

Forefoot: sagittal = parallel to long axis of metatarsal bones; axial = perpendicular to long axis of metatarsal bones; coronal = parallel to foot sole.

## Introduction

The Charcot foot has been first described in 1868 by Jean-Martin Charcot, a French pathologist and neurologist, in patients with tabes dorsalis (myelopathy due to syphilis) [1]. The detailed pathomechanisms of this disease still remain unclear: there is consensus that the cause is multifactorial and that polyneuropathy (reduced pain sensation and proprioception) is the underlying basic condition of this disease. In industrialized countries, diabetes mellitus is the main cause of polyneuropathy in the lower limb [2]—much more common than other causes like alcohol abuse or malnutrition. The prevalence of Charcot foot in a general diabetic population is estimated between 0.1 and 7.5%, but regarding diabetic patients with apparent peripheral neuropathy, this prevalence is increasing up to 35% [3]. The risk of getting a Charcot foot is not related to the type (I or II) of diabetes mellitus. The incidence of bilateral involvement of the feet has been reported between 9 and 75% [2]. Patients with Charcot foot typically present within their fifties or sixties and most of them have had diabetes mellitus for at least 10 years [2].

### Natural course of disease

**Fig. 1:**
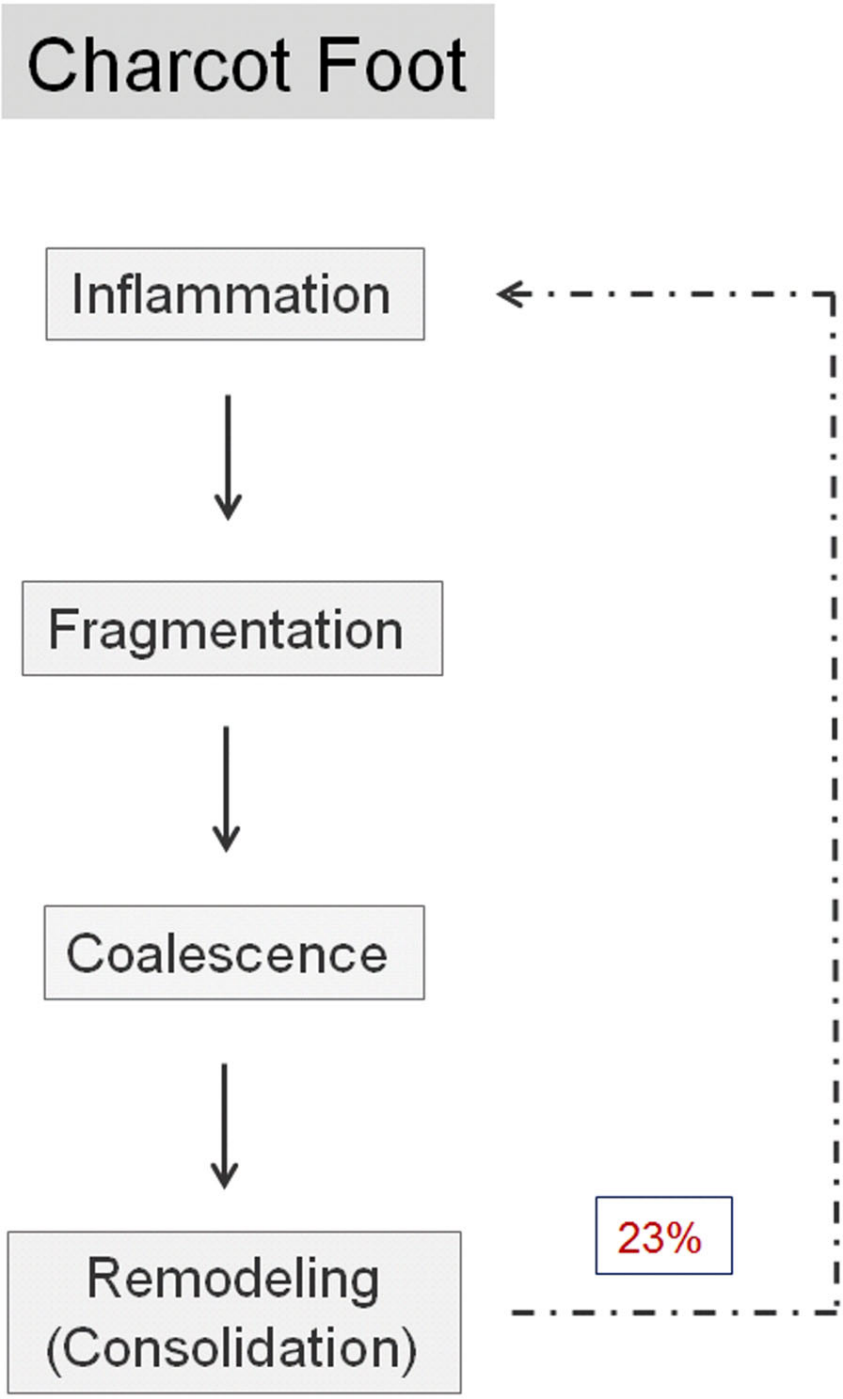
Charcot foot: natural course of disease with recurrence rates about 23%, adapted from [4–6]

**Fig. 2:**
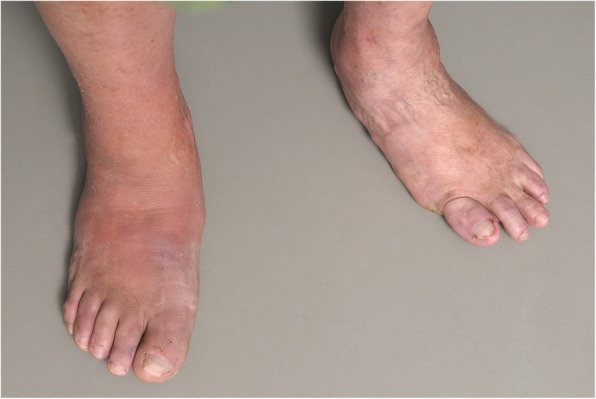
A typical Charcot foot in acute active phase: red, hot, and swollen right foot

**Fig. 3:**
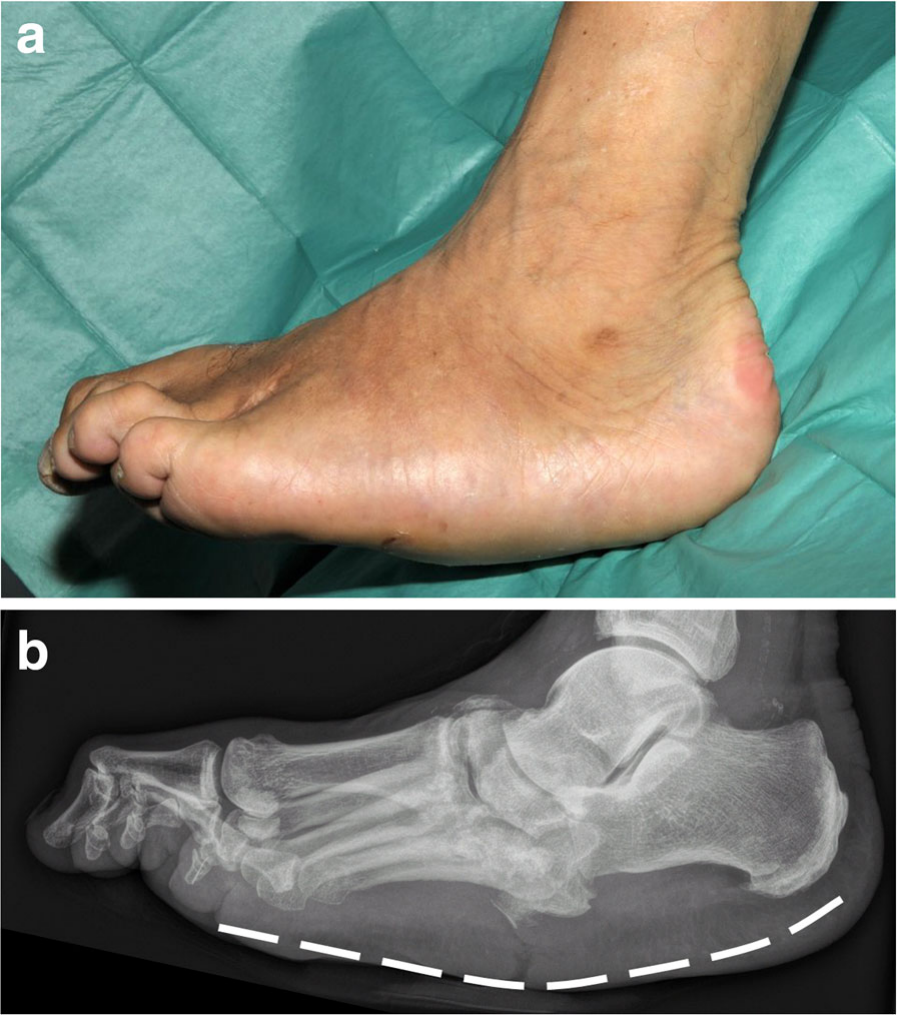
Rocker-bottom deformity: end-stage of Charcot foot. **a** Clinical image. **b** Corresponding lateral radiograph

Charcot foot is characterized by four different disease stages (Fig. 1) [7, 8], resembling active and inactive disease phases: inflammation, fragmentation, coalescence, consolidation. The disease is normally limited to a single-run through these different disease stages. The active phase is characterized by a hot, red, and swollen foot (inflammation), often without pain, due to the polyneuropathy (Fig. 2) [1]. In the active phase, the bone gets fragile due to temporary osteopenia leading to fractures, joint destructions (often Lisfranc’s joint) and collapse of the longitudinal arch of the foot [2, 8, 9]. During the less active or inactive phase, the foot is not red any more, but some soft tissue and bone marrow edema may last. Prominent osteophytes and palpable loose bodies are the consequence of a substantial joint and bone destruction followed by bony proliferations [2, 9]. The typical end-stage appearance of a Charcot foot is the so-called rocker-bottom deformity (Fig. 3).

A recent study showed that there is a risk of re-activation of a “formerly in-active” Charcot foot in about 23% within a mean interval of 27 months [4] (Fig. 1).

### Clinical stages and differential diagnoses

The (modified) Eichenholtz classification [5, 6], which relies on clinical and x-ray findings, is frequently used for clinical assessment of a suspected Charcot foot (stages 0, I, II, III, IV). Stage 0 is the ideal stage for early diagnose of a Charcot foot, but also the most difficult one for the clinician: the patients typically present with a red, swollen, warm foot, but no visible changes (yet) on radiographs. Typical differential diagnoses in this early stage include deep vein thrombosis, gout, osteoarthritis, and infection (cellulitis/osteomyelitis) [10].

### Treatment

**Fig. 4:**
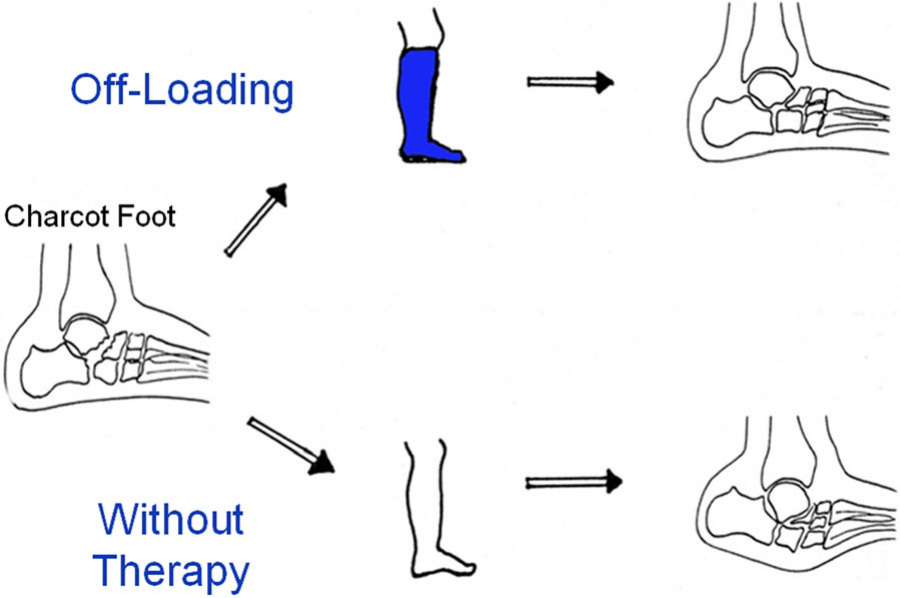
Off-loading therapy with total contact casts give the patient the chance of healing properly without debilitating deformities and with a preserved longitudinal arch

**Fig. 5:**
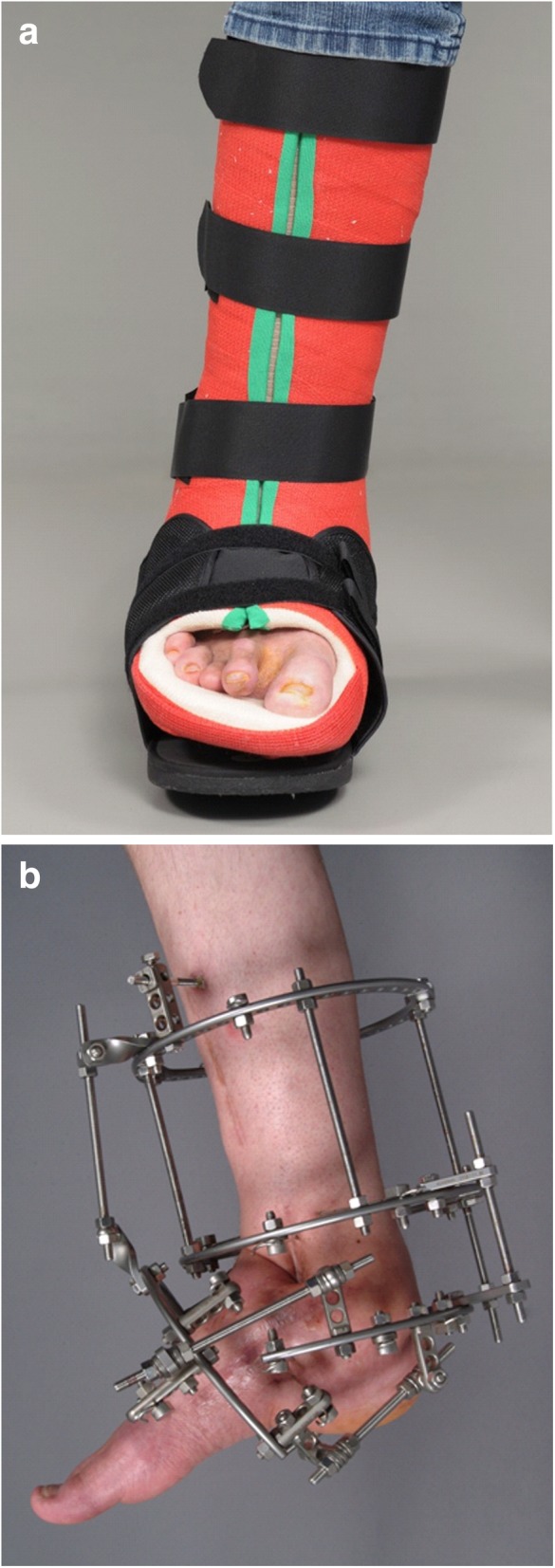
**a** Removable total contact cast used for off-loading treatment of active Charcot foot. **b** Ilizarov fixateur in a patient with Charcot foot

Current state-of-the art treatment is the off-loading of the affected foot—as soon as possible—so that the mentioned four disease stages run-through while the foot is protected from major shape changes (Fig. 4) [1]. One commonly used method is the treatment of patients with custom-made removable total contact casts (Fig. 5a) until the activity signs of the Charcot foot are significantly reduced or gone. This might take up to 18 months [4]. Establishing an early diagnosis and therefore an early off-loading treatment is crucial for the prognosis and outcome of an acute Charcot foot. The stabilization with the Ilizarov external fixator frame is considered an alternative treatment option for the off-loading [11] (Fig. 5b) in feet with complications (severe deformity or after the removal of osteomyelitic bone fragments) [12].

## Imaging findings

This review is focused on typical findings of a Charcot foot on radiographs and MR imaging since these two modalities play the most important role for disease monitoring, classification, and treatment [13].

### Classifications

The Charcot foot can be classified using various systems according to anatomical landmarks and clinical symptoms. The most common ones are the Sanders and Frykberg classification, the Brodsky classification, and the Eichenholtz-classification [5, 7, 14–17]. This review covers the Sanders and Frykberg classification in detail, because it can be used without additional clinical information.

#### Sanders and Frykberg classification

**Fig. 6:**
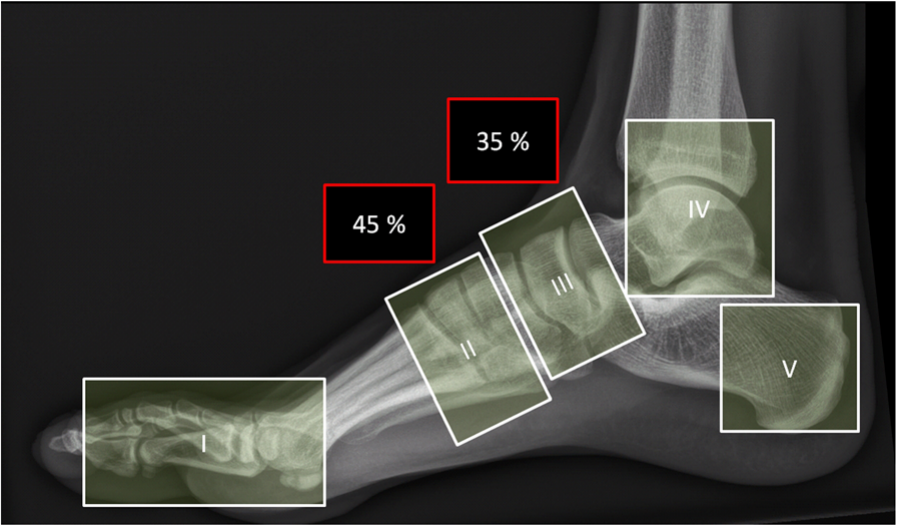
Anatomical distribution in the Sanders and Frykberg classification. Zone I: metatarsophalangeal and interphalangeal joints, zone II: tarsometatarsal joints, zone III: tarsal joints, zone IV: ankle and subtalar joints, and zone V: calcaneus

**Fig. 7:**
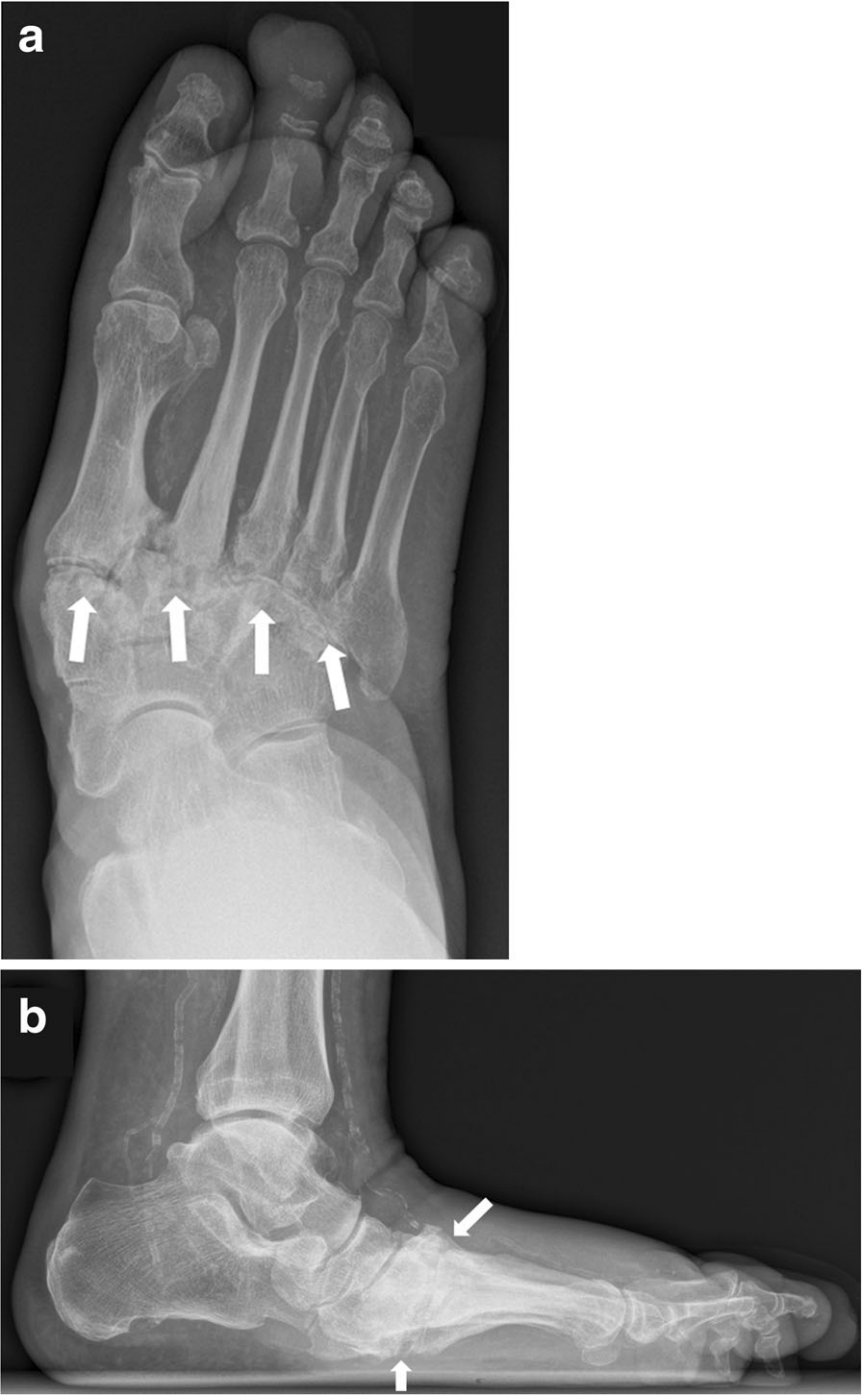
Radiographs of the right foot in dp (**a**) and lateral projection (**b**) involving zone II. Note the involvement of the tarsometatarsal articulations (white arrows) with lateral subluxation of the metatarsal bones in the Lisfranc’s joint

**Fig. 8:**
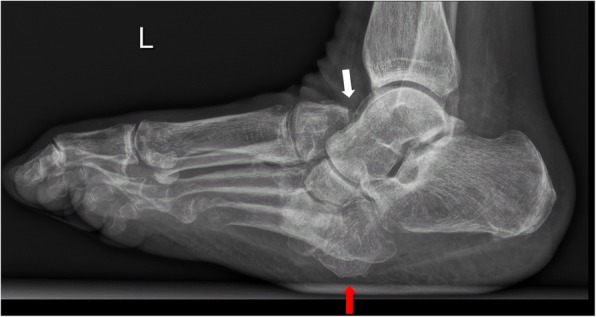
Lateral radiograph of the left foot in a patient with Charcot foot involving zone III according to Sanders and Frykberg classification (tarsal joints). The white arrow points at the typical inferior luxation of the talar head; the red arrow points at the cuboid, typically becoming the most inferior bone of the foot

Sanders and Frykberg identified five zones of disease distribution according to their anatomical location, as demonstrated in Fig. 6. Most commonly involved are zone II in about 45% and zone III in about 35% of cases [2], Fig. 7 and Fig. 8.

### Role of conventional radiographs

**Fig. 9:**
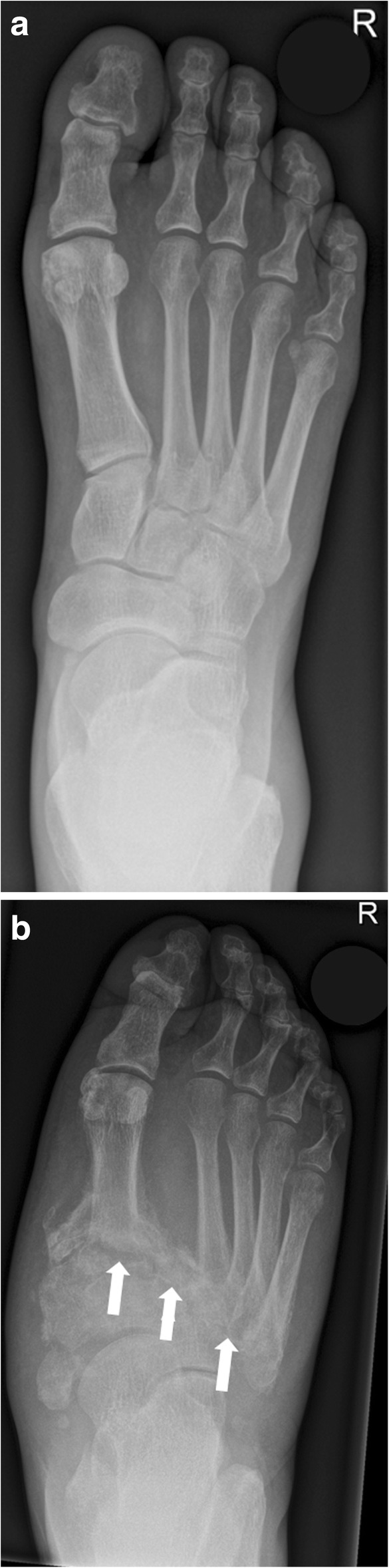
Weight-bearing radiograph in dp projection (**a** baseline, **b** 5 months later). Notice the development of fractures and subchondral cysts, erosions, joint distention, and luxation of the Lisfranc’s joint (white arrows)

Conventional radiographs of the Charcot foot are traditionally the standard imaging technique to establish the diagnosis, to stage, and to monitor the disease. The main value of plain radiographs is to assess the position of the bones to each other in general, and in particular under load (Fig. 9) [13, 18, 19].

**Fig. 10:**
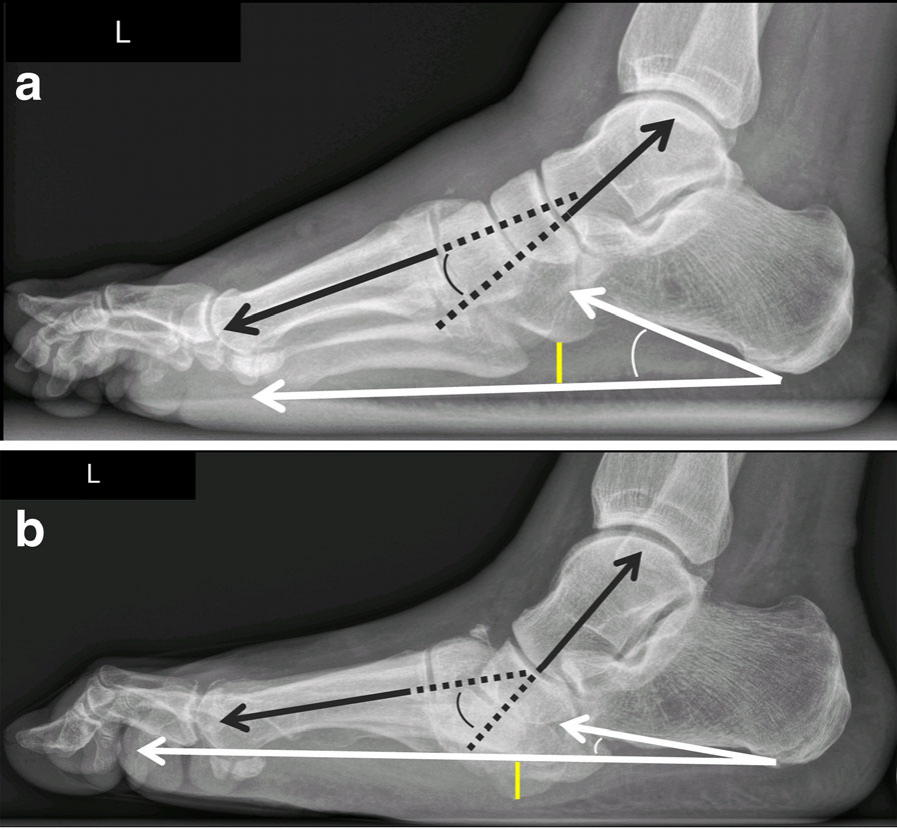
Lateral weight-bearing radiographs showing the typical course of Charcot foot disease over time (**a** baseline, **b** 10 months later). Note the continuous increase of Meary’s angle (black angle), the diminution of cuboid height, which is becoming negative (yellow distance) and the decrease of the calcaneal pitch (white angle) [19]

**Fig. 11:**
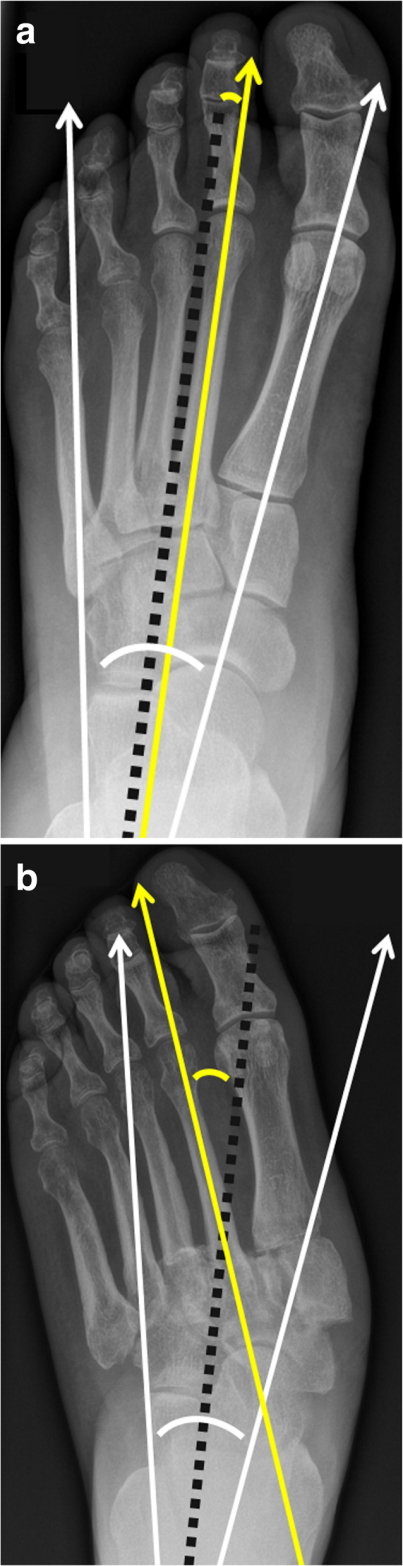
Radiograph in dp projection showing the changes in foot morphology in a typical Charcot foot patient over time (**a** baseline, **b** 10 months later). Note the increase in forefoot abduction relative to the hindfoot: The hindfoot-forefoot angle (yellow curve) is the angle between the longitudinal axis of the 2nd metatarsal bone (yellow line) and the bisection (black dotted line) of another angle (white curve), which is formed by the following two lines: the midline through the talar neck and head and a line parallel to the lateral cortex of the calcaneus (white arrows) [19]

Typical measurements on radiographs [19] help to determine the severity of deformation in a Charcot foot (especially in follow up studies), Fig. 10:

1. Meary’s angle: angle between the line originating from the center of the body of the talus, bisecting the talar neck and head, and the line through the longitudinal axis of 1st metatarsal; normal value should be around 0°.
2. Cuboid height: perpendicular distance from the plantar aspect of the cuboid to a line drawn from the plantar surface of the calcaneal tuberosity to the plantar aspect of the 5th metatarsal head. Mean normal value is about 1.2 cm above that line.
3. Calcaneal pitch: angle between a line extending from the plantar aspect of the calcaneus to the plantar surface of the 5th metatarsal head and the line extending from the most plantar portion of the calcaneal tuberosity to the most plantar portion of the anterior calcaneus [18]. Normal value lies between 20 and 30°.
4. Hindfoot-forefoot angle: Dorsoplantar (dp) radiographs can reliably show the (sub-)luxation in the Lisfranc’s joint, especially the medial aspect of the joint (Fig. 11). Dorsoplantar radiographs in follow-up studies typically show the increase in forefoot abduction relative to the hindfoot over time, the so-called hindfoot-forefoot angle (Fig. 11). Oblique conventional radiographs are superior to dp-radiographs in visualizing the lateral aspect of the Lisfranc’s joint (3rd to 5th tarsometatarsal joint).

### Role of magnetic resonance imaging

**Fig. 12:**
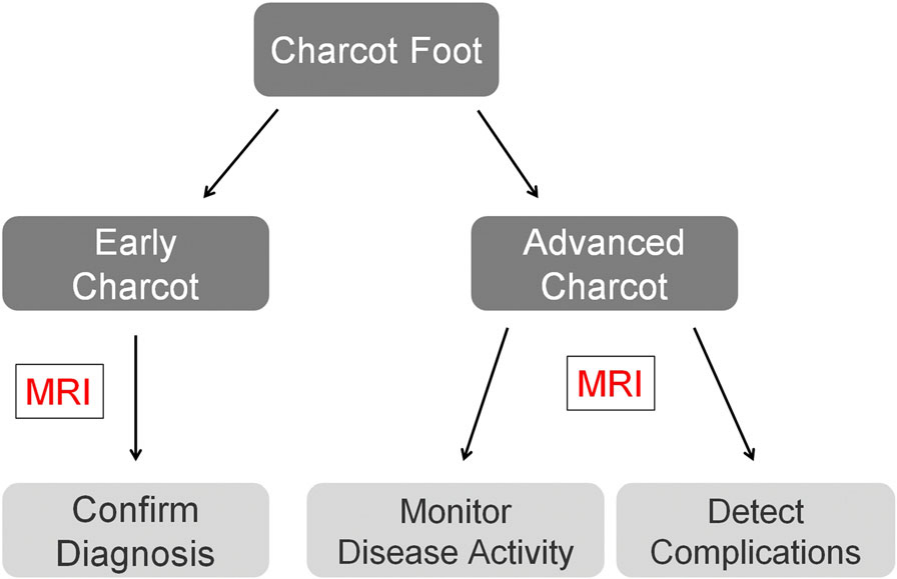
Use of MRI for diabetic patients with neuropathy in the setting of Charcot foot. Three main MRI-benefits: confirmation of diagnosis in early Charcot, monitoring of disease activity, and imaging of complications (infection/osteomyelitis)

MRI can be very helpful in order to establish an early diagnosis of Charcot foot. MRI also allows to determine the course of the healing process and the success of the off-loading treatment (monitoring: active or inactive disease). Another very significant role of MRI is its ability to further evaluate complications of a Charcot foot, in particular soft tissue infections and osteomyelitis (Fig. 12) [3, 13, 20]. In patients with contraindications for MR examination, nuclear medicine imaging can be performed (see section below: “CT and nuclear medicine imaging”).

#### MRI-protocol

**Fig. 13:**
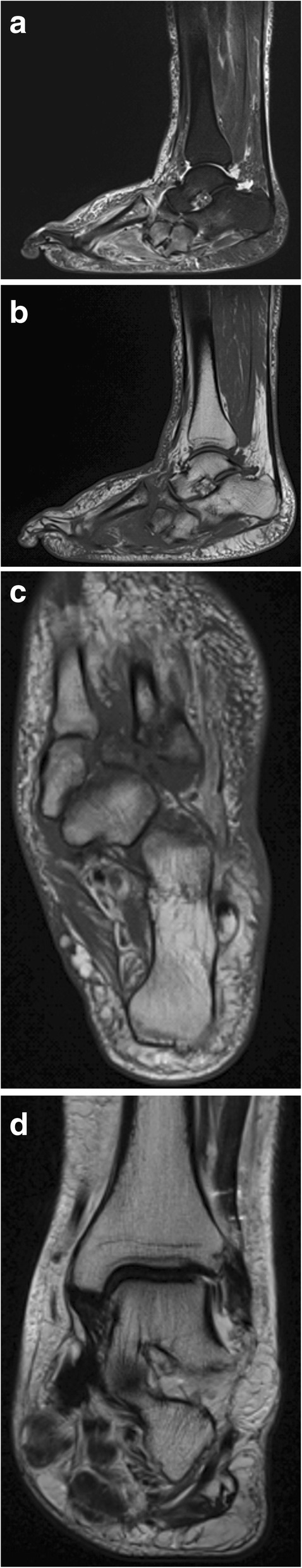
Proposed MRI-protocol for evaluation of the Charcot foot with four sequences: sagittal STIR, 3 mm, whole foot (**a**); sagittal T1, 3 mm, whole foot (**b**); transverse T1, 3 mm, hindfoot including Lisfranc’s joints (**c**); coronal T2, 3 mm, hindfoot including Lisfranc’s joints (**d**). Additional contrast media application is optional for patients with suspected infection/osteomyelitis: sagittal T1 fs 3 mm post contrast and axial T1 fs 3 mm post contrast. Note: Of course, the protocol should be extended and adapted in cases of non-Charcot-related complications, that require better spatial resolutions: e.g., additional sequences with smaller field of view, when infection at the distal toes in a diabetic foot is suspected

For Charcot foot, it is essential to use a large field of view (FoV) since the disease can affect the entire foot. It is necessary to use a fluid sensitive sequence (e.g., STIR) for assessing edema in the bone marrow and soft tissue. A classic T1 TSE (turbo spin-echo) sequence is irreplaceable to demonstrate the anatomy and the fat signal of the bone marrow. T2-weighted sequences can demonstrate the presence of subchondral cysts and help to identify fluid collections and sinus tracts [2, 3]. Axial images are useful to assess the Lisfranc’s joint disease. An MRI protocol proposal for Charcot foot evaluation is demonstrated in Fig. 13. Nephrotoxic effects of gadolinium are still controversely discussed, and almost all patients with a Charcot foot are at risk for development of renal failure (due to diabetes) [21, 22]. Therefore, the application of contrast media should be limited to patients with suspected infections (abscess collections and osteomyelitis).

#### MRI for Charcot foot diagnosis

Charcot foot cannot be diagnosed based on imaging alone and should always be interpreted in context with the clinical parameters (known polyneuropathy, red foot, and so on) [2, 23]. However, there are some typical MR imaging features for the early- and late-stage of a Charcot foot.

##### MRI for diagnosis of early-stage Charcot foot

**Fig. 14:**
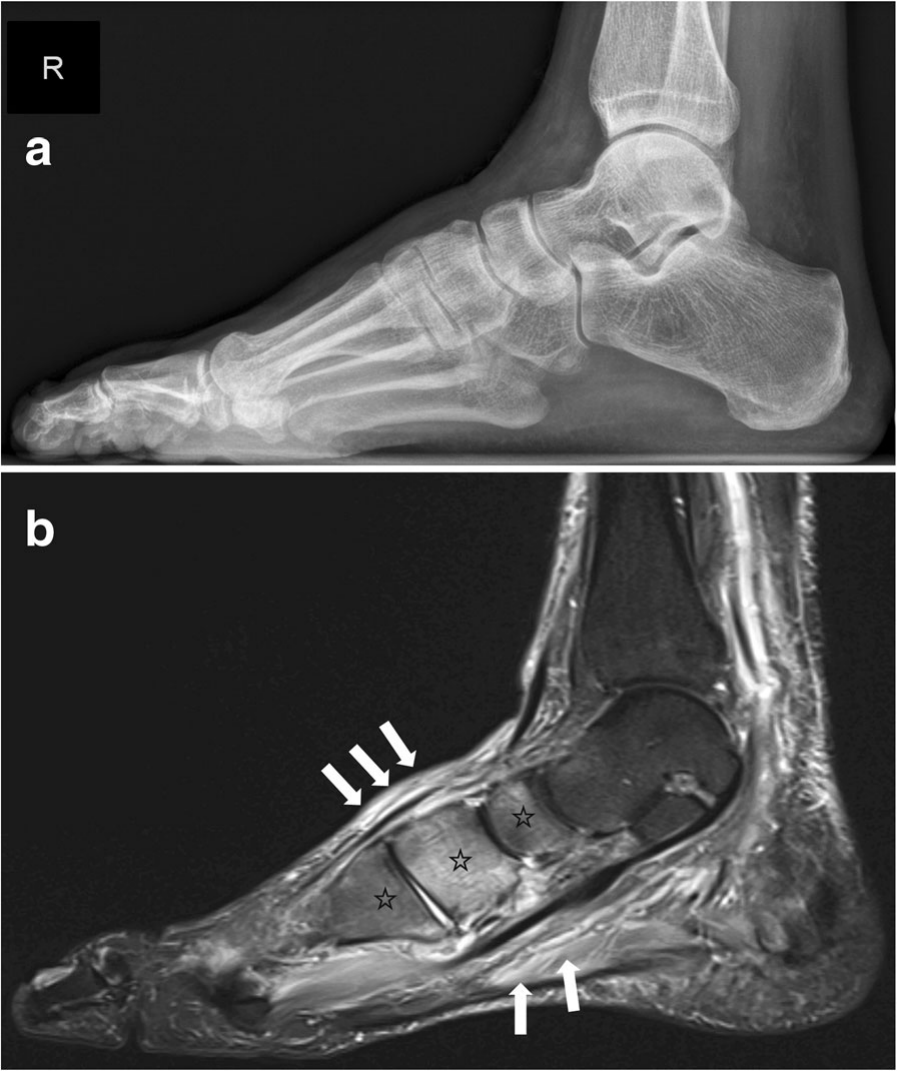
Imaging of early active Charcot foot. **a** Lateral weight-bearing radiograph showing no abnormalities. **b** Sagittal STIR-Sequence in MRI showing classic bone marrow edema in the midfoot (black asterisks) and the soft tissue and muscle edema (white arrows) in the midfoot

MRI is the best imaging modality to confirm diagnosis of suspected early active Charcot disease [24]. This may be crucial, since conventional radiographs can appear normal during very early stage of Charcot disease (Eichenholtz stage 0, Fig. 14). Early signs of a Charcot foot in MRI are bone marrow edema and soft tissue edema, joint effusion, and eventually microfractures (subchondral) [2, 25]. During early stage of Charcot foot, there are no cortical fractures and no gross deformity seen [26].

##### MRI of middle- to late-stage Charcot foot (fragmentation to consolidation)

**Fig. 15:**
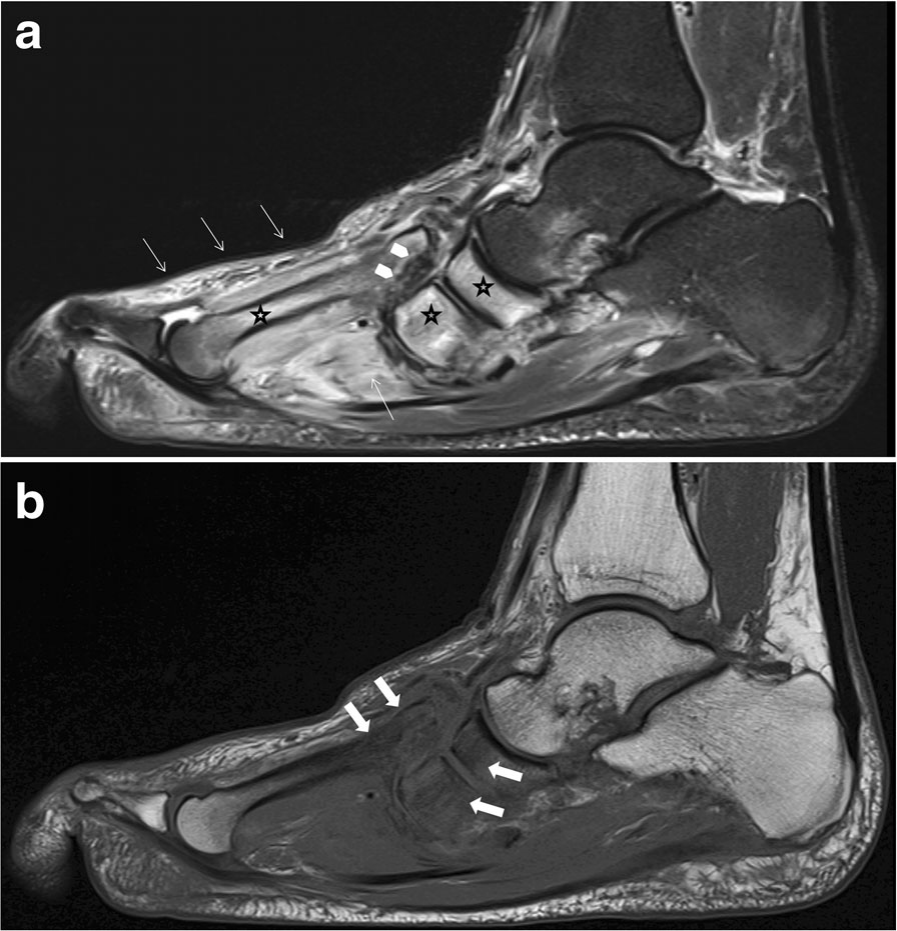
Active Charcot foot (stage of fragmentation). **a** Sagittal STIR: note the superior dislocation of the metatarsals at the level of Lisfranc’s joint (white arrow heads); massive bone marrow edema (black asterisks) in metatarsal bone, navicular bone, and cuneiform bones; and massive soft tissue edema (white thin arrows). **b** Sagittal T1: shows degree of bone destruction and fragmentation in the midfoot with huge signal drop (arrows) in the fatty bone marrow, similar to signal drops in osteomyelitis (white arrows)

**Fig. 16:**
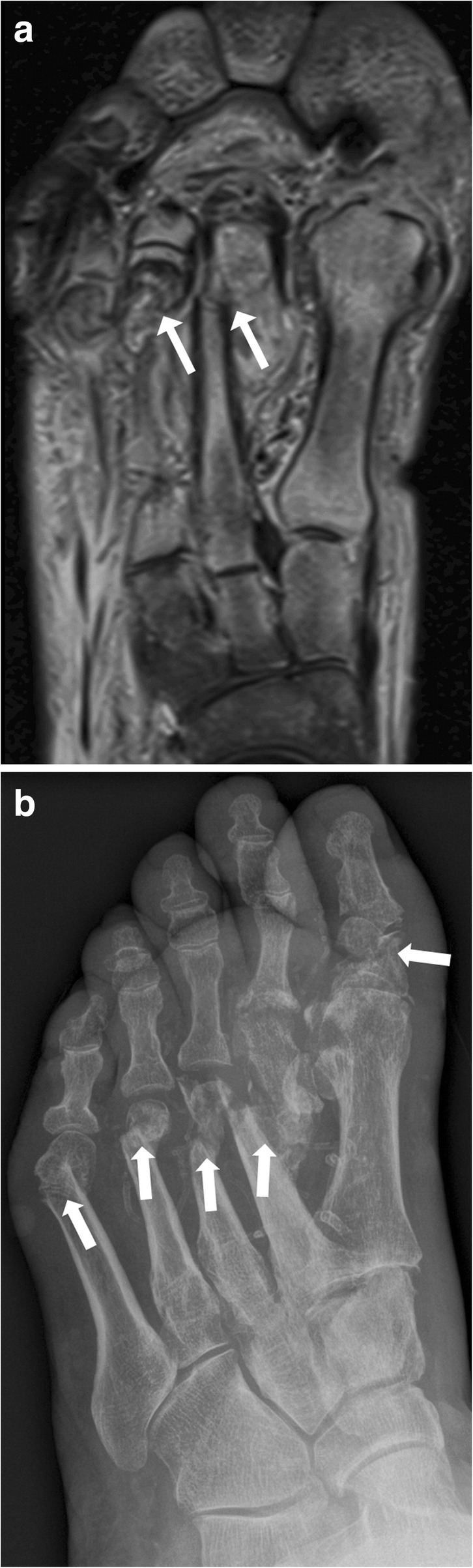
Active middle-stage disease (fragmentation) of Charcot foot demonstrating gross cortical fractures of the second to fifth metatarsal bone (white arrows) (**a** coronal STIR image of the forefoot, **b** corresponding oblique radiograph)

**Fig. 17:**
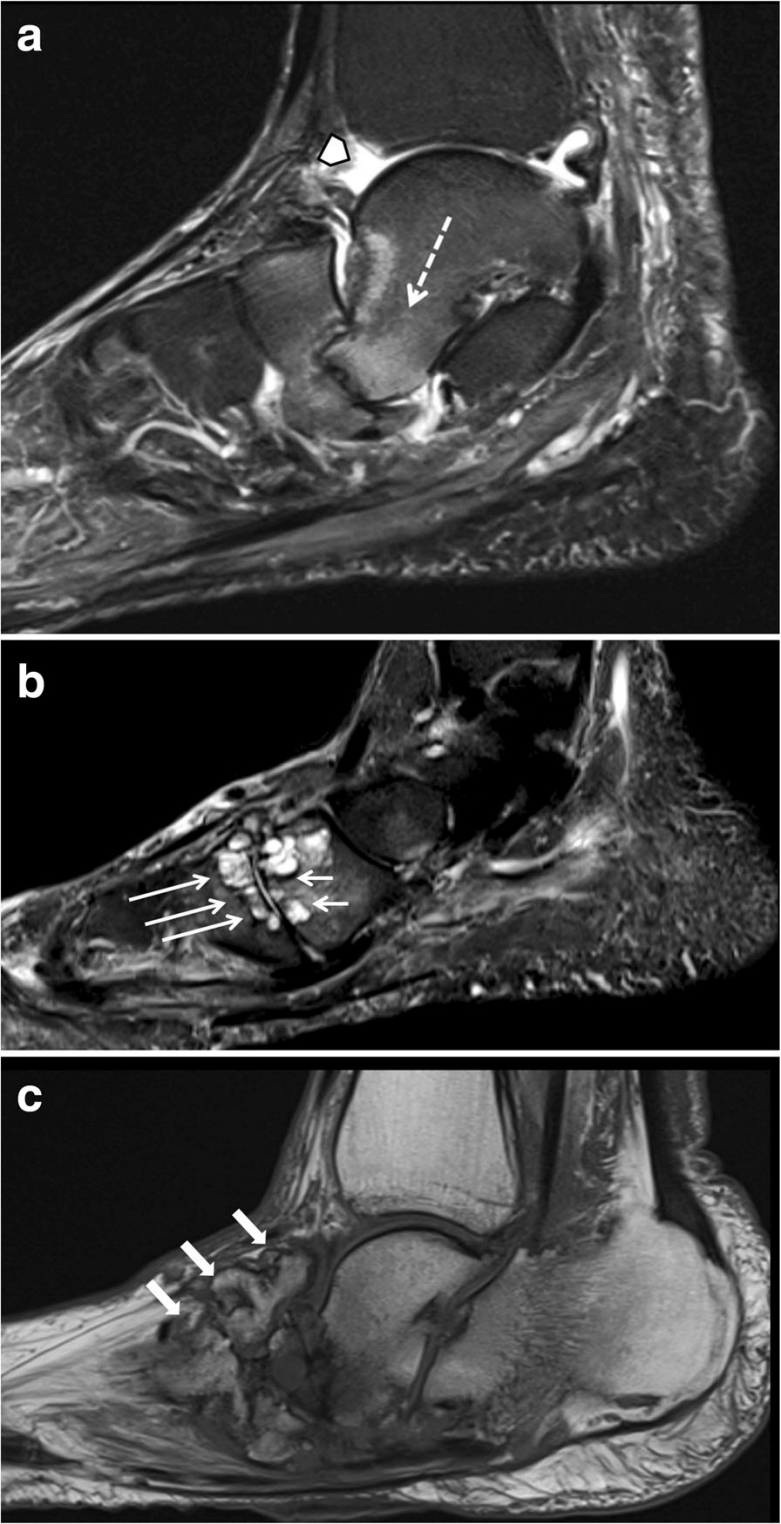
Three sagittal images of different patients showing classic features of late-stage Charcot foot. **a** (Sagittal STIR) inferior dislocation of the talar head (white arrow), effusion in the tibiotalar joint (white arrow head). **b** (Sagittal STIR) prominent subchondral cysts at the Lisfranc’s joint (white arrows). **c** (Sagittal T1) bone proliferation and debris in the midfoot (white arrows) and fragmentation of navicular bone

**Fig. 18:**
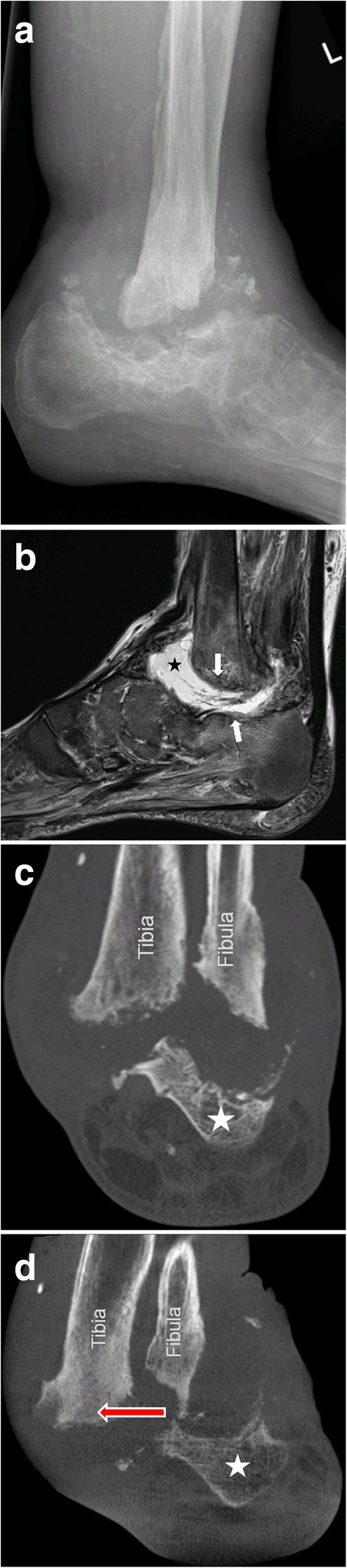
A 45-year old patient with Charcot foot and sudden shortening of the leg due to a collapse in Sanders/Frykberg zone IV (**a**). Note the huge amount of fluid (black asterisk) and debris within the impacted zone of the hindfoot (white arrows) on sagittal STIR-image (**b**). Corresponding coronal CT slice in standing position (**d**) shows medial dislocation of the hindfoot (red arrow) under weight-bearing (**d**) compared to non-weight-bearing CT (**c**). The white asterisk marks the calcaneus

Joint destruction, cortical fractures, and joint dislocations are present (Figs. 15 and 16). Bone marrow edema can be present (very common in middle-stage Charcot foot) or absent, depending on disease activity. Especially the involvement of Lisfranc’s joint leads to a typically superior and lateral dislocation of the metatarsal bones leading to a complete collapse of the longitudinal arch [2, 24, 25]. The talus head is typically tilted toward the sole of the foot (Fig. 17a), the navicular bone typically dislocates into a medial and superior position, often with fractures and fragmentation. Prominent well-marginated subchondral cysts are a typical feature of the chronic Charcot foot (Fig. 17b). Bone proliferation and sclerosis, debris, and intraarticular bodies can occur (Fig. 17c) [2, 26]. Fluid collections surrounding destructed joints may be huge (Fig. 18).

#### Monitoring of disease activity with MRI

**Fig. 19:**
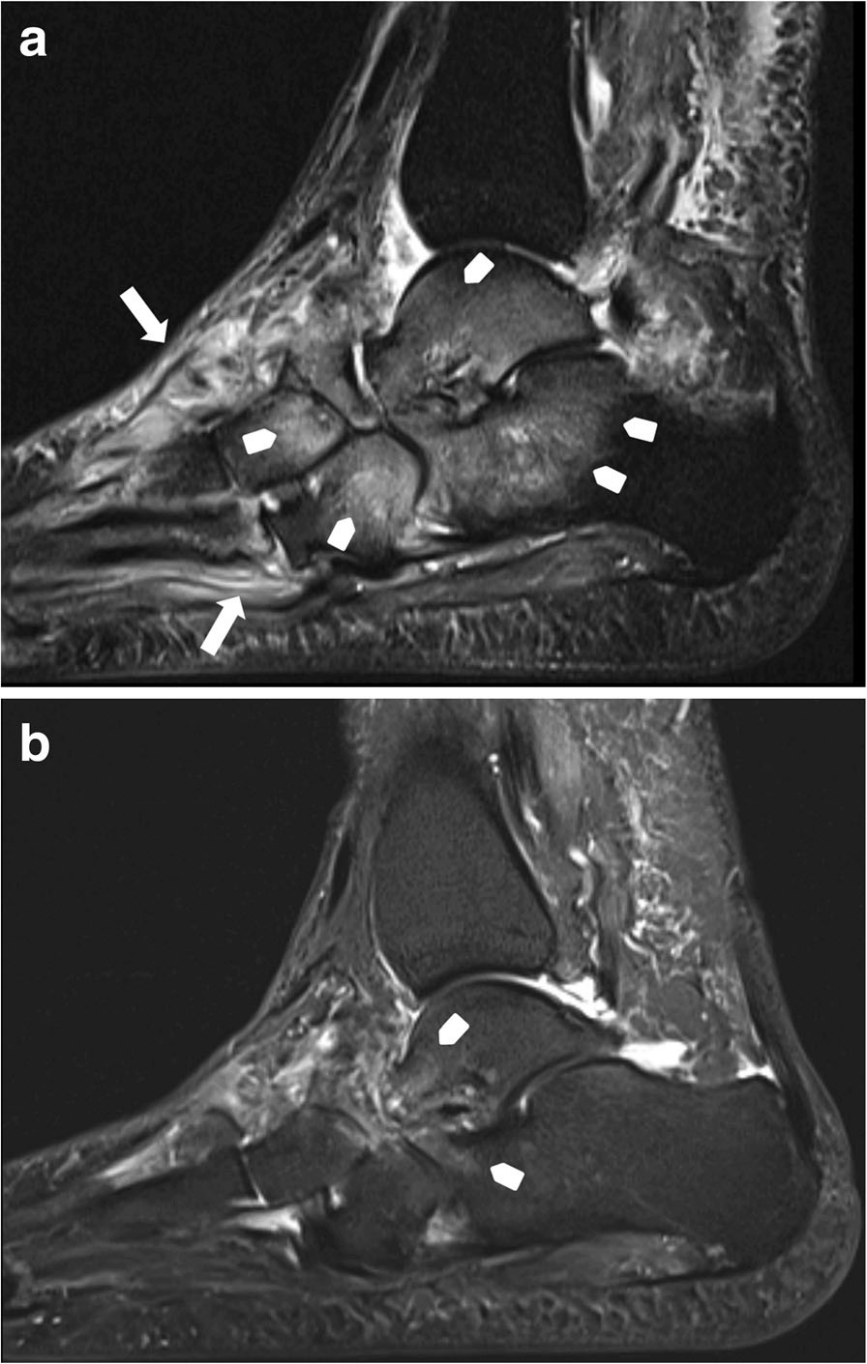
Before off-loading therapy (**a** sagittal STIR, **b** sagittal T1): active stage of Charcot disease with a significant amount of bone marrow edema (white arrow heads) and soft tissue edema (white arrows) (**a**). Also note the subluxation at the Chopard’s joint with downward tilt of the talar head (**b**) 7 months after a consequent off-loading therapy with a total contact cast: note the almost complete disappearance of bone marrow edema (white arrow heads) and soft tissue edema on sagittal STIR sequence

MRI is the best imaging modality to monitor the disease activity. As long as a significant amount of bone marrow edema is seen on MRI, consequent off-loading therapy with removable total contact casts has to be continued [27]. After a significant decrease or complete disappearance of bone marrow edema, the cast can be removed, and an orthopedic shoe adapted (Fig. 19).

#### MR-imaging of complications: infection/osteomyelitis

**Fig. 20:**
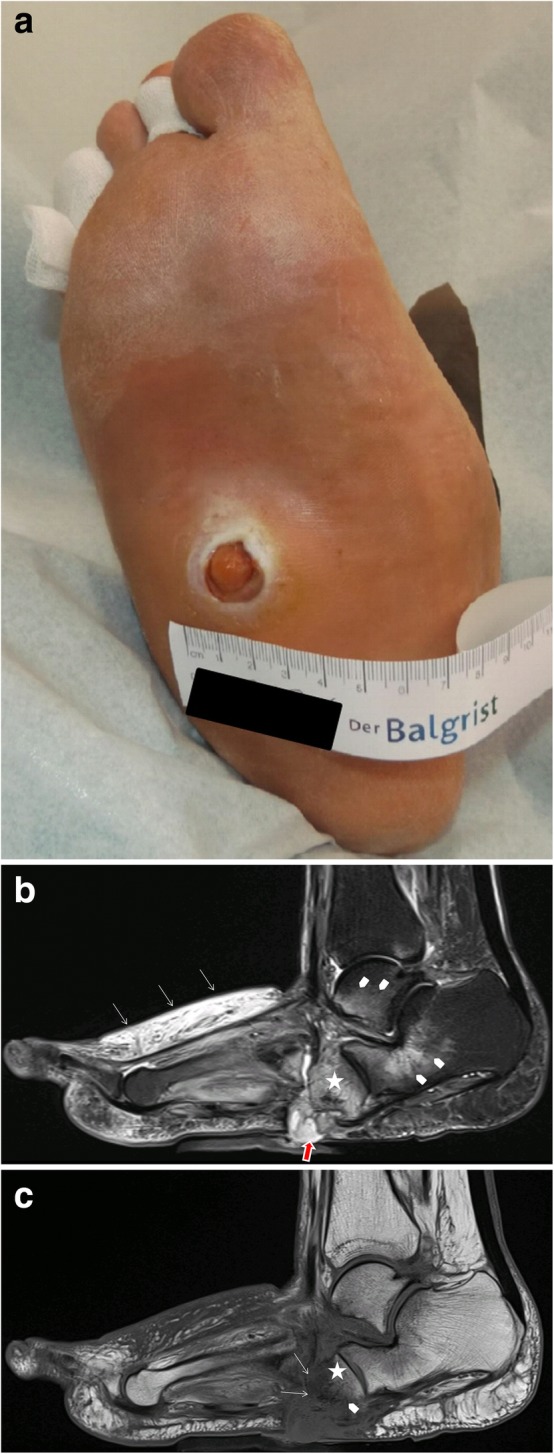
Patient with ulceration (**a**) at the sole of the foot directly beneath the cuboid bone as a typical complication of rocker-bottom deformity of the foot. MRI with sagittal STIR sequence (**b**) demonstrates contiguous spread of infection from the skin, forming a sinus tract (red arrow) to the cuboid bone (asterisk) and bone marrow edema due to active Charcot disease (arrow heads). Sagittal T1-weighted sequence shows focal replacement of fatty bone marrow signal within the cuboid bone (**c**), representing osteomyelitis

In Charcot foot, the cuboid bone typically becomes the most inferior bone in the foot [3] (Fig. 20). Due to the resulting changes in pedal shape, the foot is prone to extensive callus formation, blisters, and ulcerations, especially plantar to the cuboid bone (Fig. 20c). This may lead to soft tissue infections and osteomyelitis (Fig. 20a, b) [2].

MRI has a high diagnostic accuracy in diagnosing osteomyelitis of the foot, with a high sensitivity (77–100%) and a high specificity (80–100%) [24]. MRI has a very high negative predictive value (98%): if there are no signs of osteomyelitis on MRI, osteomyelitis can practically be excluded [28].

**Fig. 21:**
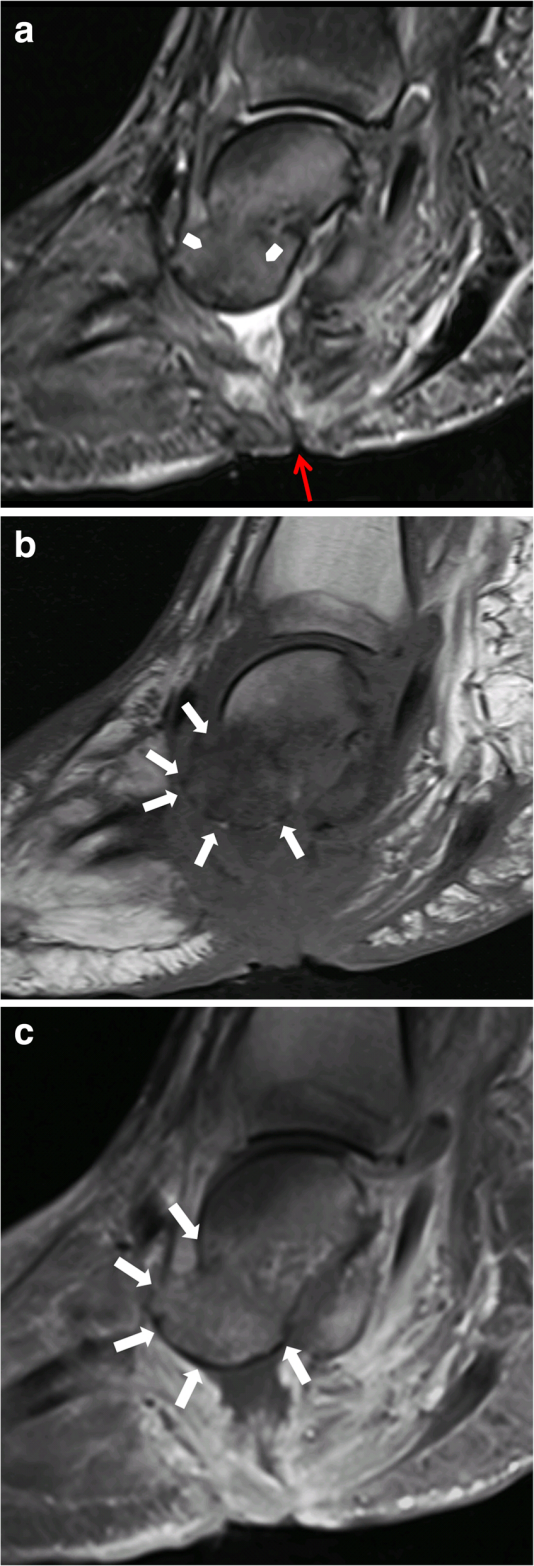
MRI of a Charcot foot complicated with osteomyelitis. **a** Sagittal T1. **b** Sagittal STIR. **c** Sagittal T1 fat sat after contrast administration. Skin ulceration and sinus tract extending from the skin to the talar bone are present, showing a direct spread of infection (red arrow) (**a**). Diffuse bone marrow alteration is present within the talus. Note the disappearance of bony contours in the sagittal T1-weighted image (white arrows in **b**) and the reappearance of the bone structures after contrast administration (white arrows in **c**) demonstrating the “Ghost sign,” which is described in many reviews as pathognomonic for osteomyelitis in Charcot foot [20]. However, up to now, there is no study published evaluating the accuracy of this sign

However, discriminating an active Charcot foot from acute osteomyelitis remains challenging [25]. Both entities have similar image characteristics like bone marrow edema, soft tissue edema, joint effusions, fluid collections, and contrast enhancement in bone marrow and soft tissues. Even the degree of signal drop in T1 sequences might be quite similar in both conditions (Figs. 15 and 20). However, there are some imaging features (listed in Table 1, Fig. 21) that may help to find the correct diagnosis.

**Table 1:** MRI features for differentiating an active Charcot foot from osteomyelitis. Information collected from Ahmadi et al. 2006 [25], Donovan and Schweitzer 2010 [29], Ergen et al. 2013 [2], Johnson et al. 2009 [28], Martín Noguerol et al. 2017 [30], Mautone and Naidoo 2015 [24], Schoots et al. 2010 [3], and Toledano et al. 2011 [20]

	Active Charcot foot	Osteomyelitis
Location of bone marrow abnormality (edema shown in fluid sensitive sequences, and reduction of fatty bone marrow shown in T1 sequences)	○ Pattern tends to be periarticular ○ Usually involves several joints and bones (mostly tarso-metatarsal joints and metatarsophalangeal joints)	○ Tendency to involve a single bone with diffuse marrow involvement ○ Usually affects weight-bearing surfaces of the toes, metatarsal heads, calcaneus, malleolus, and a special area in Charcot: cuboid (in rocker-bottom deformity) ○ Develops almost exclusively by continuous spread of infection from skin ulcerations and sinus tracts
Sinus tracts	o Usually not present	o Often present
Skin ulceration (technician should mark the exact ulcer location)	o Can be present	o Often present o Often relationship to sinus tract
Fluid collections	o Present o Usually smaller than in case of infection, unless sinus tract is present	o Present o Usually larger than in active Charcot foot, unless a sinus tract exists over which the collection is drained (paradoxical decrease of size of fluid collection) o Diffusion-weighted imaging (DWI) might help in differentiation abscesses from non-infected fluid collections
Subcutaneous fat	o Dorsal often with edema, plantar often normal	o Often disappears due to presence of cellulitis
Subchondral cysts	o Typical image feature in chronic Charcot foot o The presence of subchondral cysts indicates the absence of infection	o Tendency to disappear in case of infection/osteomyelitis o Best recognized if regular previous follow-up studies are present, which demonstrate the disappearance of the cysts
Intraarticular bodies	o The presence of intraarticular bodies indicates the absence of infection	o Often disappear in the setting of infection due to dissolution or obscureness by surrounding inflammation
“The ghost sign”	o Negative: a neuropathic joint without infection will not demonstrate the “ghost sign” because the bones are definitely destroyed and will look destroyed on all sequences	o Positive: bones that “disappear” on T1-weighted images and “reappear” (outline of the bone becomes visible again) after contrast administration (or on T2-weighted images)—suspicious of osteomyelitis—Fig. 21

#### Advanced MR-imaging techniques

Diffusion-weighted imaging may contribute in the detection and extension of osteomyelitis: pure edema does not show diffusion restriction, whereas the presence of pus and inflammatory cells in infection leads to restricted diffusion with lower ADC-values than in pure edema [31]. Dynamic contrast enhancement (DCE)-perfusion may help in the discrimination between viable tissue and necrosis. Furthermore, the enhancement pattern in DCE-perfusion seems to be different between osteomyelitis and osteoarthropathic changes, increasing the potential of differencing lesions with bone marrow edema [30].

### CT and nuclear medicine imaging

During early-stage Charcot foot, CT does not play an important role for imaging since bone marrow and soft tissue changes can be better visualized using MRI [2]. However, CT may be used in later-stage Charcot foot for better visualization of bony proliferations and consolidation, or for surgery planning and treatment monitoring in patients with Ilizarov fixation [2]. Furthermore, CT and PET-CT may be used as an alternative cross-section imaging tool in patients with contraindications for MR examination (pacemaker, severe claustrophobia, etc.). PET-CT allows the quantification of the inflammatory process in all stages of Charcot foot and allows to follow-up its evolution over time: recent research showed that PET-CT may be of additional help for evaluation of treatment duration in addition to MR imaging [32].

Furthermore, nuclear medicine imaging may be of important value in non-conclusive cases with suspected infection of a Charcot foot: a recent meta-analysis compared MRI, FDG–PET-CT, and white blood cell scintigraphy [33]. The authors concluded that despite all of these modalities having a similar sensitivity for detection of osteomyelitis in Charcot foot, the nuclear imaging methods show a higher specificity [33]. However, all nuclear medicine imaging methods are more expensive than MRI and result in radiation exposure to the patient.

## Conclusion

The Charcot foot is a rare disease, associated with polyneuropathy, in industrialized countries most commonly seen in the long-term diabetic population. The radiologist plays an important role in the management of this disease. Therefore, it is important to be familiar with the typical imaging characteristics of the Charcot foot and to consider this diagnosis in a proper clinical setting. Recognizing this disease in early stages prevents a delayed onset of an appropriate therapy and helps minimizing the disability of these patients.

Although radiographs are important to assess the position of the bones to each other in general, and in particular under load, MRI is the method of choice not only in establishing an early diagnosis but also in monitoring the course of the disease activity and in diagnosing infectious complications.

## Data Availability

Data sharing is not applicable to this article as no datasets were generated or analyzed. A different version of this article was presented as an educational poster at the ESSR (European Society of Musculoskeletal Radiology) congress 2018 in Amsterdam.

## Abbreviations

ADC: Apparent diffusion coefficient
CT: Computed tomography
Dp: Dorsoplantar
FDG: Fluorodesoxyglucose
FoV: Field of view
MR: Magnetic resonance
MRI: Magnetic resonance imaging
PET: Positron emission tomography
STIR: Short tau inversion recovery
